# Exploiting Transient Protein States for the Design of Small-Molecule Stabilizers of Mutant p53

**DOI:** 10.1016/j.str.2015.10.016

**Published:** 2015-12-01

**Authors:** Andreas C. Joerger, Matthias R. Bauer, Rainer Wilcken, Matthias G.J. Baud, Hannes Harbrecht, Thomas E. Exner, Frank M. Boeckler, John Spencer, Alan R. Fersht

**Affiliations:** 1 MRC Laboratory of Molecular Biology, Francis Crick Avenue, Cambridge CB2 0QH, UK; 2 Molecular Design and Pharmaceutical Biophysics, Institute of Pharmaceutical Sciences, Eberhard Karls Universität Tübingen, Auf der Morgenstelle 8, 72076 Tübingen, Germany; 3 Department of Chemistry, School of Life Sciences, University of Sussex, Falmer, Brighton, East Sussex BN1 9QJ, UK

**Keywords:** p53, mutant rescue, drug design, molecular chaperone, transient protein states

## Abstract

The destabilizing p53 cancer mutation Y220C creates an extended crevice on the surface of the protein that can be targeted by small-molecule stabilizers. Here, we identify different classes of small molecules that bind to this crevice and determine their binding modes by X-ray crystallography. These structures reveal two major conformational states of the pocket and a cryptic, transiently open hydrophobic subpocket that is modulated by Cys220. In one instance, specifically targeting this transient protein state by a pyrrole moiety resulted in a 40-fold increase in binding affinity. Molecular dynamics simulations showed that both open and closed states of this subsite were populated at comparable frequencies along the trajectories. Our data extend the framework for the design of high-affinity Y220C mutant binders for use in personalized anticancer therapy and, more generally, highlight the importance of implementing protein dynamics and hydration patterns in the drug-discovery process.

## Highlights

• Surface crevice in the p53 cancer mutant Y220C targeted with small molecules
• Numerous high-resolution Y220C-ligand structures determined
• Transiently open hydrophobic subpocket identified
• Structures provide a framework for the design of potent binders for cancer therapy

## Teaser

Joerger et al. determined structures of the p53 cancer mutant Y220C in complex with stabilizing small molecules, revealing that some of these molecules target a transiently open subpocket. These data provide a framework for the design of potent molecules for personalized anticancer therapy.

## Introduction

The rational design of potent, pharmacologically active chemical probes to target disease-related proteins is aided by a detailed understanding of the target protein structure, its conformational flexibility, available molecular interactions, and solvation patterns (Bissantz et al., 2010, Teague, 2003). Protein molecular dynamics (MD) simulations may help to identify induced-fit mechanisms of ligand binding, modulate potential interaction networks, and reveal surface properties not observed in the available crystal structures. For several pharmaceutically relevant targets, MD simulations suggest the presence of cryptic, only transiently open binding pockets or subpockets (Bernini et al., 2014, Kokh et al., 2013, Wassman et al., 2013). Using a single static crystal structure of a ligand-free protein for rational drug design may miss intrinsic flexibility of binding sites. Insights into the structural plasticity of a binding pocket can, however, be obtained from an ensemble of different crystal structures, for example by comparing the structure of the target protein in different crystal forms or different molecules within an asymmetric unit, or by analyzing binding-pocket variations in structures with different ligands. The latter approach has recently led to the identification of transient conformational states of the N-terminal domain of the MDM2 protein that can be exploited for the design of inhibitors of the p53-MDM2 interaction (Bista et al., 2013).

The tumor-suppressor protein p53 is a prime target for therapeutic intervention in cancer. It is inactivated in virtually every tumor either through missense mutation in the p53 gene or as a result of perturbations in its regulatory pathways, for example by overexpression of its negative regulators MDM2 and MDMX (Joerger and Fersht, 2010, Khoo et al., 2014, Uversky et al., 2014, Wade et al., 2013). Most oncogenic p53 mutations are located in the DNA binding domain (DBD) and either cause loss of essential contacts with DNA (contact mutants) or perturb the structure of this domain (structural mutants) (Joerger and Fersht, 2007). Because of its low intrinsic stability, the DBD is very susceptible to inactivation by destabilizing mutations, causing the protein to rapidly unfold, aggregate, and lose its transcriptional activity (Bullock et al., 2000, Wilcken et al., 2012b). In principle, it should be possible to rescue the tumor-suppressor function of these structural mutants with small molecules that selectively bind to the folded but not the denatured state of the mutant protein, thereby shifting its conformational equilibrium and stabilizing an active, wild-type-like conformation. The cancer mutation Y220C, which accounts for an estimated 100,000 new cancer cases per year worldwide (Joerger and Fersht, 2007), is an ideal test case for this rescue strategy with molecular chaperones. Mutation of a tyrosine on the surface of the protein to a smaller cysteine residue opens up an extended but narrow crevice that can be targeted by small molecules (Joerger et al., 2006). By using virtual screening, we discovered a carbazole derivative, PhiKan083 (**1**), which binds to the Y220C pocket, raises the melting temperature of the protein, and slows down its rate of aggregation (Boeckler et al., 2008). We subsequently identified additional, chemically diverse molecules that bind to the mutation-induced surface crevice in the Y220C mutant (Basse et al., 2010, Wilcken et al., 2012a) and synthesized a molecule that is biologically active in cancer cell lines with homozygous Y220C mutation (Liu et al., 2013), establishing the proof of concept that functional restoration of mutant p53 by small molecules is a viable strategy.

Here, we report high-resolution crystal structures of the Y220C mutant bound to a series of structurally diverse small-molecule compounds that were identified through screening of fragment libraries or developed through structure-based design strategies. Combined with MD simulations, these data provide insights into the structural plasticity of the mutation-induced surface crevice, revealing a transiently open hydrophobic subpocket, and pinpoint key interactions for the design of binders with increased potency.

## Results and Discussion

### Overall Architecture of the Y220C Binding Pocket

**Figure 1:**
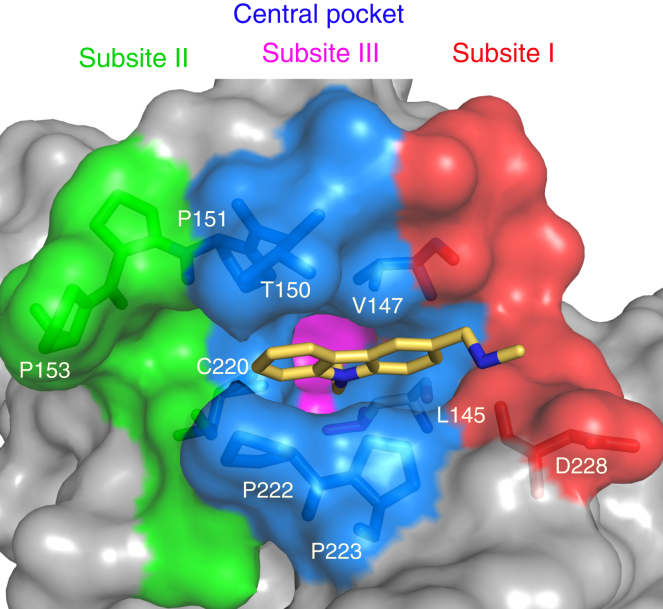
Mutation-Induced Surface Crevice in the p53 Cancer Mutant Y220C Molecular surface representation of the binding pocket in complex with the carbazole derivative PhiKan083 (**1**) (PDB: 2VUK). The binding pocket can be formally subdivided into a central cavity (blue) and subsites I (red), II (green), and III (magenta). In the ligand-free structure and the complex with PhiKan083, subsite III is blocked by the Cys220 side chain.

The Y220C mutation creates an extended but narrow crevice on the surface of the p53 protein (Joerger et al., 2006). This crevice is flanked by two proline-rich loops, S3/S4 and S7/S8, and has its greatest depth at the mutation site. The binding site can formally be subdivided into a central cavity and three subsites, I, II, and III (Figure 1). PhiKan083 (**1**), the first structurally characterized Y220C binder, is sandwiched between several prolines (Pro151, Pro222, and Pro223) and Val147 in the central cavity, with the ethyl group serving as a hydrophobic anchor. It extends into subsite I via its methylamine linker, where it forms a hydrogen bond with the backbone oxygen of Asp228. This shallow, solvent-exposed subsite is largely polar, making its targeting by small molecules difficult. A narrow channel on the opposite end of the central cavity leads into subsite II, where several prolines, including Pro153, provide a hydrophobic interaction surface, with several backbone oxygens lining this hydrophobic patch (Cys220, Pro151, and Pro152). As we outline in detail in the following, there is an additional, transiently open subsite III at the bottom of the central cavity, which is essentially modulated by the conformational state of Cys220.

### Crystal Structures of Y220C-Ligand Complexes

**Figure 2:**
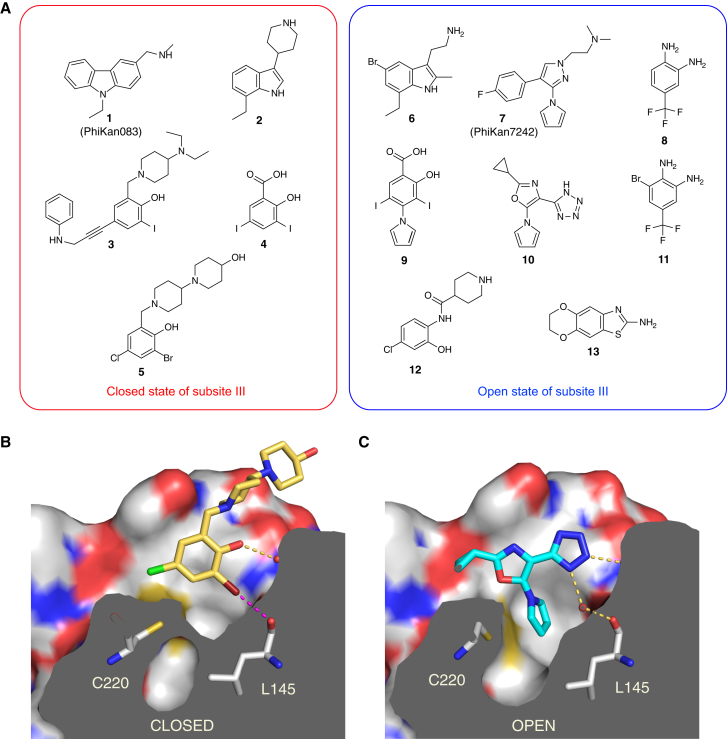
Small-Molecule Stabilizers of the p53 Cancer Mutant Y220C (A) Chemical structures of compounds binding to the mutation-induced surface crevice in p53-Y220C. Compounds are grouped according to the conformational state of the Y220C pocket (closed or open subsite III) in the structure of the complex. (B) Molecular surface of the Y220C pocket (cross section) in complex with **5**. The Cys220 sulfur atom faces the solvent and blocks subsite III. The bromine atom forms a halogen bond (broken magenta line) with the backbone oxygen of Leu145 with near ideal bond geometry (distance = 3.0 Å, σ-hole angle [C_ar_-Br···O] = 174°). (C) Molecular surface of the Y220C pocket (cross section) in complex with **10**. In this case, Cys220 adopts an alternative conformation with the sulfur pointing toward the interior of the protein, thereby opening up a hydrophobic subpocket (subsite III), which is occupied by the pyrrole moiety of the ligand. Yellow broken lines in (B) and (C) indicate water-mediated hydrogen bonds. Water molecules are shown as small red spheres.

From screening of fragment libraries combined with structure-based design, we have identified a series of chemically diverse compounds that bind to the Y220C mutant with varying affinities and have determined their binding mode by X-ray crystallography (Figure 2; Tables 1 and 2). Together with our previously published Y220C-ligand structures, they provide snapshots of the dynamic nature of the binding site and highlight key interactions for the design of potent binders. The first striking aspect upon analysis of these structures is that the various small molecules sample two different conformational states of the binding pocket that are essentially modulated by Cys220 (Figure 2). Rotamer switch of the side chain of Cys220 from *trans* to gauche(−) conformation opens up a small hydrophobic pocket at the bottom of the binding site (subsite III). In the “closed” state with *trans* conformation of the Cys220 side chain, which is observed in the ligand-free structure, the sulfur is solvent exposed, whereas it contacts several hydrophobic residues in the “open” state of the pocket with gauche(−) conformation of Cys220. Rotation of the cysteine side chain is generally also accompanied by small changes in the backbone conformation. Conformational flexibility in the subsite III region is not restricted to Cys220 but also extends further into the hydrophobic core of the β-sandwich region. Both Val157 and Ile232 adopt alternative side-chain conformations, depending on the bound ligand, which is indicative of the dynamic nature of this region of the binding pocket. Alternative conformations are also observed for the side chain of Thr230.

### Ligand Binding Modes and Conformational Selection

**Figure 3:**
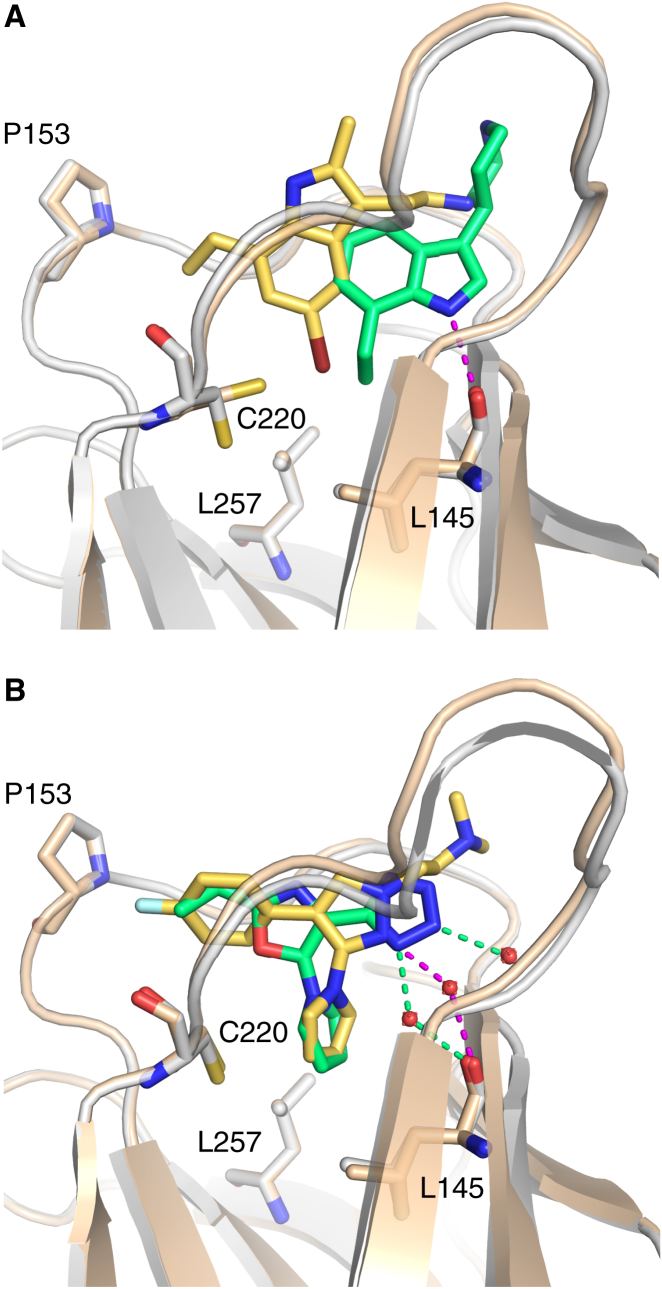
Differential Conformational Selection of p53-Y220C Ligands (A) Superposition of the structures of two indole derivatives, **2** (green ligand, salmon protein chain) and **6** (yellow ligand, gray protein chain), in complex with p53-Y220C. Despite sharing a common scaffold, the two compounds adopt different binding modes and target a different conformational state of the Y220C pocket. The hydrogen bond between **2** and the backbone oxygen of Leu145 is highlighted with a dashed magenta line. (B) Superposition of the structures of **7** (yellow ligand, gray protein chain; PDB: 3ZME) and **10** (green ligand, salmon protein chain) in complex with p53-Y220C. Both compounds target the same conformational state of the Y220C pocket and bind to the open state of subsite III via a pyrrole moiety. Interactions with selected structural water molecules are highlighted with dashed lines.

The structures of Y220C with different indole-based fragments provided insights into the structural plasticity of the binding site. The binding mode of fragment **2** mimics that of the carbazole-based PhiKan083 (**1**, Figure 3A). The indole ring binds to the center of the binding pocket and is sandwiched between Pro223 on one side and Val147/Pro151 on the other side. The indole nitrogen forms a hydrogen bond with the backbone carbonyl of Leu145, thereby displacing a structural water molecule. The ethyl group serves as a hydrophobic anchor at the bottom of the binding pocket. The piperidine moiety reaches into the solvent and contributes little to the overall binding. Interestingly, additional methyl/bromo substituents on the indole scaffold and a shorter, more flexible linker to the protonated amine in fragment **6** completely alter the binding mode of the fragment within the Y220C pocket (Figure 3A). It binds to the “open” state of subsite III. The central indole moiety sits upright within the pocket, occupying different parts of the central cavity than fragment **2**, and the bromo moiety points into subsite III where it forms hydrophobic interactions. This example illustrates both the conformational plasticity of the binding pocket and the fact that the same scaffold, depending on its substitution pattern, can select different conformational states and target different subsites of the pocket.

The 2,4,5-substituted oxazole **10** and the 1,3,4-substituted pyrazole **7** (PhiKan7242) represent another interesting compound pair with regard to their conformational selection. In this case, both molecules bind to the “open” conformation of the binding pocket and target subsite III via a pyrrole moiety (Figure 3B). Despite the different position of the central ring system in the binding pocket, the pyrrole moieties occupy essentially the same position and engage in extensive hydrophobic interactions within the transiently open subsite III (e.g., with Leu145, Val147, Cys220, and Leu257). In both cases, the second hydrophobic substituent (4-fluorophenyl/cyclopropyl) points toward subsite II. The tetrazole in **10** is solvent exposed and interacts with two conserved structural water molecules. We have recently shown that PhiKan7088, an analog of **7**, is biologically active in cancer cell lines expressing the Y220C mutant (Liu et al., 2013), which highlights the potential of ligands that effectively target subsite III for future cancer therapy.

### Exploiting the Transiently Open Subsite III for Ligand Design

**Figure 4:**
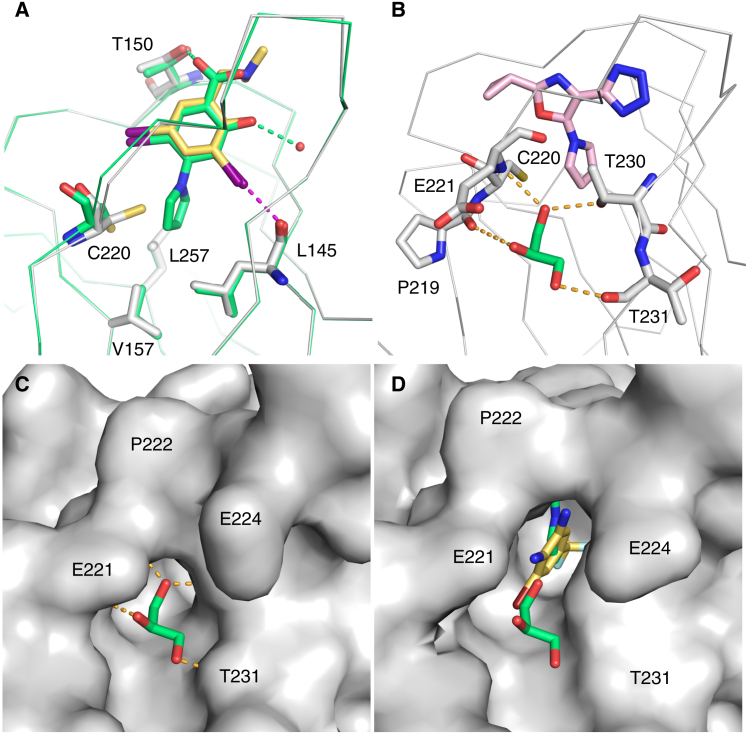
Targeting the Transiently Open Subsite III (A) Superposition of the binding modes of **9** (green) and its precursor chemotype without pyrrole moiety (yellow ligand, gray protein chain; PDB: 4AGL) shows that the pyrrole moiety binds to the open state of subsite III, without significant effect on the overall binding mode of the central scaffold. The protein chains are shown as Cα traces, and the small molecules and selected protein residues as stick models. The broken magenta line indicates the halogen bond formed by one of the iodine substituents with the backbone oxygen of Leu145. Hydrogen bonds of **9** with Thr150 and a structural water molecule are highlighted with green broken lines. (B) Structure of the Y220C-**10** complex with bound glycerol. As in (A), the protein is shown as a Cα trace with selected residues highlighted as stick models. **10** is shown in pink. A glycerol molecule trapped from the soaking buffer, shown as a green stick model, binds close to the pyrrole moiety of **10** but outside the Y220C pocket. Hydrogen bonds between protein and glycerol are highlighted with orange broken lines. (C) Molecular surface representation of the glycerol binding site in the Y220C-**10** complex. (D) Molecular surface representation of the Y220C mutant in complex with **11**. Fragment **11** (yellow stick model) has a unique binding mode, and binds between the binding sites of the pyrrole moiety of **10** and the glycerol molecule (both shown as green stick models). As a result, binding of **11** opens up and stabilizes a small channel that links subsite III and the glycerol binding site.

Analysis of the binding modes of the iodophenol series (e.g., **3**) and molecule **7** suggested merging of these compound series. We therefore designed hybrid compounds to evaluate the potential gain in affinity by introducing a pyrrole moiety that targets the open state of subsite III. Based on this rational design strategy, we synthetically introduced a pyrrole in position 4 of the 3,5-diiodo-salicylic acid scaffold **4** (*K*_D_ = 820 μM; ligand efficiency [LE] = 0.35 kcal/mol per non-hydrogen atom). The resulting compound **9** (*K*_D_ = 21 μM; LE = 0.38) displayed a 40-fold increase in affinity and maintained high LE, thus highlighting an important design criterion based on subsite III exploration to develop potent ligands (Table 2). Determination of the crystal structure of **9** in complex with Y220C confirmed that the pyrrole moiety binds to the open state of subsite III as anticipated. Interestingly, the presence of the pyrrole did not significantly affect the binding mode of the central scaffold, which retained its interaction network, including the halogen bond to the carbonyl oxygen of Leu145 (Figure 4A).

Intriguingly, the crystal structure of the Y220C-**10** complex revealed a bound glycerol molecule close to the pyrrole moiety in subsite III. This glycerol, which was trapped from the soaking buffer, forms hydrogen bonds with the main-chain oxygen of Pro219, the main-chain nitrogen of Glu221, the side chain of Thr230, and the main-chain oxygen of Thr231, as well as a number of structural water molecules (Figure 4B). The closest distance between the glycerol and the pyrrole moiety of **10** is 3.3 Å, but there is no channel connecting both binding sites. In this context, the binding mode of the small fragment **11** is of particular interest (Figure 4D). It is buried deeply within subsite III and forms hydrophobic interactions with Leu145, Val157, Leu257, Phe109, and a hydrogen bond with the main-chain oxygen of Pro222. The protein backbone around Pro222 is significantly displaced upon ligand binding (up to 3.5 Å), which further increases the depth and width of subsite III. This movement connects subsite III with the glycerol binding site, indicating that the Y220C mutant protein may sample a conformation with a transiently open channel in solution (Figures 4C and 4D). Future design strategies may therefore exploit the structural plasticity of the binding pocket, and introduce subsite III moieties that probe the conformational ensemble of this region and potentially extend into the glycerol binding site.

### The Role of Structural Water Networks within the Binding Pocket

**Figure 5:**
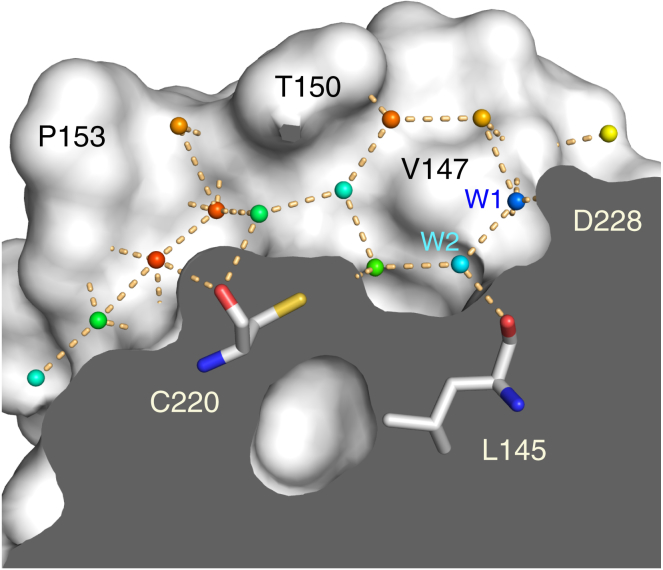
Hydration Pattern of the Y220C Pocket Molecular surface representation of a cross section of the mutation-induced surface crevice in p53-Y220C (PDB: 2J1X, chain A). Structural water molecules inside the pocket are shown as spheres and colored according to their *B* factors in a rainbow-colored gradient from blue (*B* factor = 15 Å^2^) to red (*B* factor = 50 Å^2^). The *B* factors over all water molecules in chain A of the structure range from 8 to 53 Å^2^. Particularly stable water molecules are labeled W1 and W2. Orange broken lines indicate hydrogen bonds.

Determining the hydration pattern of protein binding sites aids the understanding of ligand binding and improving ligand affinity (Bissantz et al., 2010). There is a network in the ligand-free crystal structure of the Y220C mutant of several water molecules in the mutation-induced cavity (Figure 5). Two adjacent water molecules, which are also present in the wild-type structure, exhibit on average lower *B* factors than the remaining waters in the cavity and form a well-defined hydrogen-bond network (water molecules W1 and W2 in Figure 5). One of these waters (W1) sits at the entrance toward subsite I and connects two carbonyl oxygens from different loops of the binding pocket (Val147 and Asp228). The second water (W2) lies more centrally within the pocket, where it forms a hydrogen bond with the Leu145 backbone oxygen and Thr230. Depending on the chain in the crystal structure, the hydrogen bond is formed with either the side-chain hydroxyl or the main-chain nitrogen. In the wild-type structure, structural water W2 is further stabilized via a hydrogen bond with the hydroxyl group of Tyr220. It is displaced in most ligand complexes, except for **10** and **7**, where the small molecules form a hydrogen bond with the structural water (Figure 3B). Upon water displacement, most ligands made polar interactions with the Leu145 backbone, through either a hydrogen or halogen bond (see Figures 2A and 3A), mimicking the water-mediated interaction and compensating for the enthalpic cost of water displacement. W1 was retained in many ligand structures and often formed part of the polar ligand interaction network. Although there are several accessible backbone carbonyl oxygens within subsite II that can act as hydrogen-bond acceptors (e.g., backbone of Pro152, Pro153, and Cys220), the solvent structure in this region was generally less well defined in the available crystal structures.

### Targeting Subsite II

**Figure 6:**
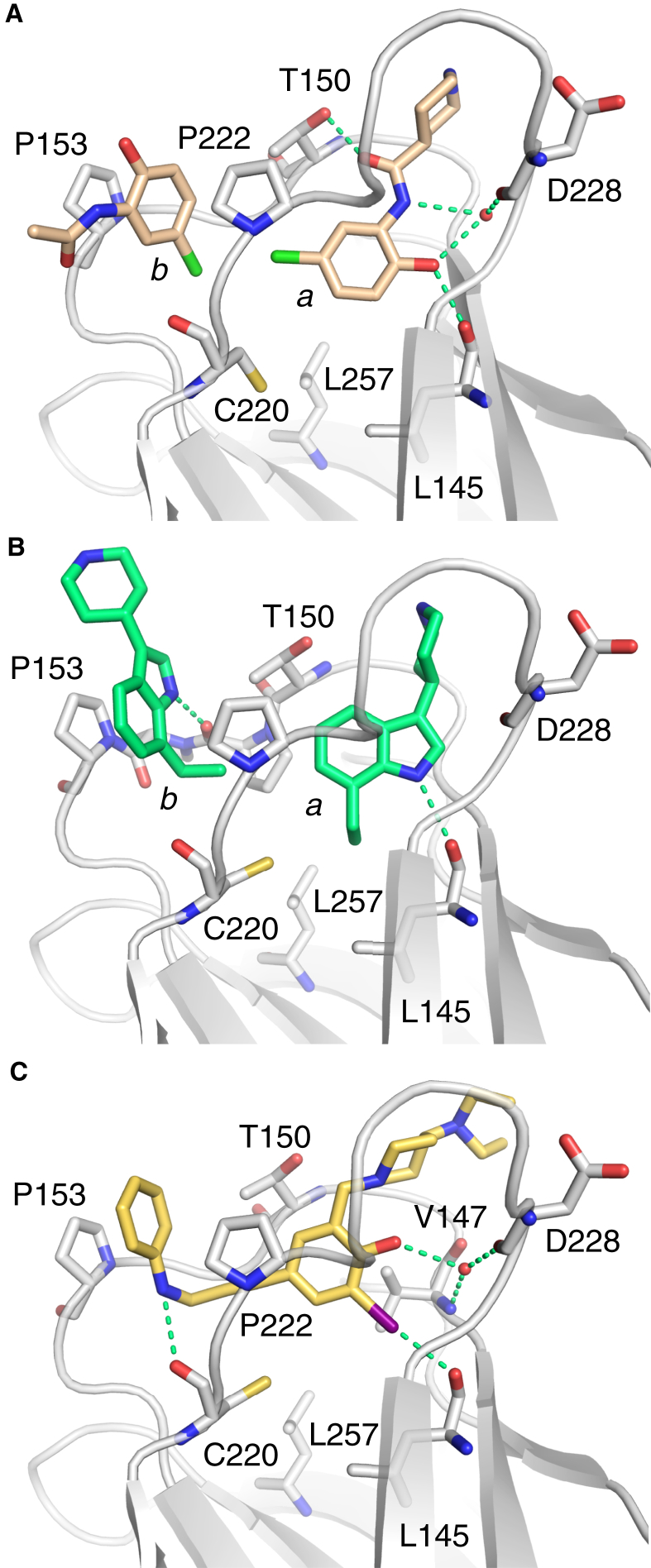
Targeting Subsite II with Aromatic Systems Structure of the Y220C mutant in complex with the chlorophenol **12** (A) and the indole **2** (B), showing that in both cases two small molecules bind to the Y220C pocket, one at the center (a) and one in subsite II (b). The binding mode of the molecules in subsite II is reminiscent of that of the aniline moiety of molecule **3** (C), which also packs against Pro153 (PDB: 4AGQ). Polar interactions (hydrogen and halogen bonds) between the small molecules and the protein or conserved structural water molecules are highlighted with green broken lines. All three structures are shown in approximately the same orientation.

For several aromatic fragments we detected two ligand molecules bound in close proximity within the Y220C binding pocket: one at the center of the binding site and another in subsite II. The electron density for the second molecule was generally much weaker, indicating partial occupancy because of lower affinity for this secondary binding site. An example is compound **12**. Binding of **12** induced a rotamer switch of Cys220 (Figure 6A). In the primary binding mode, the chlorophenol moiety sits deep inside the central cavity, with its hydroxyl group forming hydrogen bonds with the main-chain oxygen of Leu145 and the conserved structural water molecule W1 between the backbone carbonyl of Asp228 and Val147. The chlorine faces the narrow hydrophobic channel toward subsite II. The amide group accepts a hydrogen bond from Thr150 with its carbonyl oxygen and donates a hydrogen bond to the structural water W1 via its amide NH. The piperidine moiety protrudes into the solvent, similarly to its position in the Y220C-**2** complex. A second molecule of **12** binds to subsite II where the aromatic ring packs against Pro153 and Pro222, and the chlorine substituent faces the hydrophobic channel connecting subsites II and III. This binding mode is reminiscent of that observed for the previously published dianiline **8** (Basse et al., 2010). Interestingly, a second molecule binding to subsite II was also observed for indole **2** (Figure 6B). Here, the indole ring is also engaged in hydrophobic interactions with Pro153/Pro222 and, additionally, forms a hydrogen bond with the backbone carbonyl of Pro151. A common feature of these substituted ring systems is that they are electron rich, combining the appropriate shape complementarity, hydrophobic interactions, and CH-π interactions with Pro153/Pro222. Introducing a benzene moiety in subsite II via an acetylene linker was also crucial for improving the affinity of the iodophenol series, e.g., in compound **3** (Figure 6C) (Wilcken et al., 2012a). In addition, Cys220 and Pro151 provide hydrogen-bond acceptors in an ideal orientation for hydrogen-bond formation. Taken together, these data show that subsite II and the channel leading into it are poised for interaction with appropriately substituted aromatic ring systems that combine significant electron density on the ring with a favorable hydrogen-bond donor/acceptor pattern (e.g., with Cys220 and Pro151).

### Molecular Dynamics Simulation of the Y220C Binding Pocket

**Figure 7:**
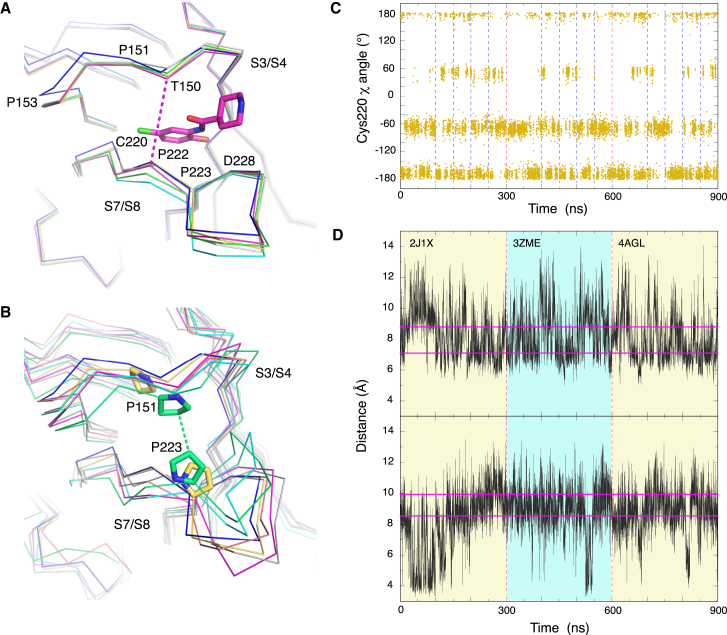
Conformational Flexibility of the Y220C Pocket (A) Superposition of the binding pocket in different crystal structures, showing shifts in the S7/S8 loop upon ligand binding that modulate the width of the binding pocket. Cα traces are shown for: ligand-free Y220C mutant (PDB: 2J1X) chains A (blue) and B (yellow), Y220C-**12** complex chain B (magenta), Y220C-**10** complex chain B (green), Y220C-7 complex chain B (cyan), and Y220C-**1** complex chain B (gray; PDB: 2VUK). Bound molecule **12** is shown as a stick model. The distance between the Cα atoms of Thr150 and Pro222 in the Y220C-**12** complex is highlighted with a magenta broken line. (B) Superposition of representative structures from major clusters along the MD trajectory onto chains A (blue) and B (yellow) of the ligand-free crystal structure. The gray and magenta Cα traces are representative structures of the two largest clusters with an open pocket, comprising more than half of all structures along the trajectory. The green structure represents an ensemble of structures with a partial hydrophobic collapse of the pocket via interactions between Pro151 and Pro223. The close distance between the Cδ atoms of Pro151 and Pro223 in this structure is highlighted with a green broken line. The view is the same as in (A). (C) Side-chain conformations of Cys220 (χ_1_ angle) along the MD trajectories. The conformation fluctuates between different rotamers corresponding to an open and close state of subsite III. Broken lines indicate starting points of different MD simulations, and those in red a new starting structure. (D) Distance between the Cα atoms of Thr150 and Pro222 (top), and between the Cδ atoms of Pro151 and Pro223 (bottom) as a function of time over all simulations. The solid magenta lines indicate the largest and smallest distances observed in the various Y220C crystal structures. Starting and end points of the various simulations are the same as in (C).

We performed MD simulations of the Y220C structure to obtain further insights into the dynamics of the binding pocket. Simulations were run for a total of 900 ns with three different starting structures (300 ns each): unbound Y220C (PDB: 2J1X), Y220C-PhiKan784 complex (PDB: 4AGL; closed subsite III) and Y220C-**7** complex (PDB: 3ZME; open subsite III). The ligands were removed before the start of the simulations. Structures along the trajectory were clustered based on the conformation of the Cys220 region as well as the overall architecture of the binding pocket. As expected, the pocket exhibited significant plasticity in these simulations. We observed both open and closed states of subsite III, independent of the starting structure, with two major side-chain conformations of Cys220, with χ_1_ angles (N-C_α_-C_β_-S_γ_) of about −60° for rotamer I (gauche(−) = “g−”) and 180°/−180° for rotamer II (*trans* = “t”), respectively (Figure 7C). In addition, there was a third Cys220 rotamer with a χ_1_ angle of about 60° (gauche(+) = “g+”), not observed in any crystal structure, which occurred less frequently along the trajectory. The population of these rotamer clusters over all simulations was about 38% (rotamer cluster I), 48% (rotamer cluster II), and 14% (rotamer cluster III). This distribution is close to published generalized rotamer statistics (e.g., DYNAMEOMICS) (Kehl et al., 2008). The fact that Cys220 conformations corresponding to both open and closed states of subsite III were populated with comparable frequency along the trajectory indicates a low energy barrier for the Cys220 side-chain rotation in the binding pocket, suggesting that it also fluctuates between alternative conformations in solution.

The width of the binding pocket varies significantly in the different Y220C crystal structures (Figure 7A). The observed distance between the Cα atoms of Thr150 and Pro222 at the rim of the surface crevice, for example, ranges from 7.1 to 8.8 Å, depending on the ligand bound. In the wild-type structure (PDB: 2XWR; Natan et al., 2011), this distance is 6.7 Å in chain A and 6.9 Å in chain B. Modulation of this distance in the Y220C-ligand complexes is largely due to a movement of the S7/S8 loop, whereas the shorter S3/S4 loop comprising Thr150 is more rigid and shows only minor shifts upon ligand binding. Both loops are not directly involved in crystal contacts. In the simulations, however, there were substantial fluctuations in both loops, with a general movement of the S3/S4 loop toward the center of the pocket (Figures 7B and 7D). Pro151, for example, showed a tendency to collapse into the pocket and interacted with Pro223 in some structures along the trajectory (especially in the first 100-ns simulation with PDB: 2J1X as a starting structure), which essentially blocked parts of the central cavity. In the remainder of the simulations, the pocket remained largely open. Overall, the simulations highlight the highly dynamic nature of the binding pocket in the absence of stabilizing ligands and support the notion that there are transiently open subpockets in solution. In the wild-type structure, S3/S4 and S7/S8 loop movements are restricted by the side chain of Tyr220 (Basse et al., 2010).

### Conclusions

The crystal structures of the p53 cancer mutant Y220C bound to stabilizing small molecules provided novel insights into key protein-ligand interactions, hydration patterns, and conformational states of the binding pocket. Importantly, we have identified a transiently open subpocket (subsite III), which is blocked by the Cys220 side chain in the crystal structure of the ligand-free mutant. Targeting this hydrophobic site with a pyrrole moiety resulted in substantial gains in affinity. The structural data and associated structure-activity relationships provide the blueprint for the design of high-affinity binders, for example by merging existing core scaffolds, or modifications thereof, with optimized subsite II and subsite III moieties. Such high-affinity ligands may act as potent anticancer agents and provide valuable chemical probes for studying p53 function in living cells.

More generally, our data highlight the usefulness of crystallographic validation of ligand binding modes early in the drug-discovery process. Screening of fragment libraries is perfectly suited for mapping the conformational ensemble of a binding site and for identifying cryptic subpockets, as shown here for the p53 Y220C mutant. Such a strategy allows targeting of transient protein states and increases the chances of finding ligands with new chemotypes and physicochemical properties that would not have been discovered with docking or rational drug discovery based solely on a single crystal structure of the apo protein.

## Experimental Procedures

### Chemical Compounds

Compounds **2** and **12** were bought from Enamine, compound **5** from Chembridge (custom-made synthesis contract), compound **6** from Vitas-M Laboratory, and compound **11** from Sigma-Aldrich. Synthesis of compounds **9** and **10** will be described elsewhere.

### Binding Assays

Dissociation constants for the binding of ligands to the p53 Y220C mutant were determined by either nuclear magnetic resonance spectroscopy or isothermal calorimetry as described by Wilcken et al. (2012a).

### Structure Determination of Y220C-Ligand Complexes

Crystals of p53C-Y220C were grown at 18°C using the sitting-drop vapor diffusion technique as described by Joerger et al. (2006). Crystals were soaked for 3–6 hr in a 30–40 mM solution of compound in cryo buffer (19% polyethylene glycol 4000, 20% glycerol, 10 mM sodium phosphate [pH 7.2], 100 mM HEPES [pH 7.2], and 150 mM NaCl). In case of poor ligand solubility, saturated solutions were used for soaking. X-Ray data were collected at 100 K at the beamlines I02, I03, and I04 of the Diamond Light Source (Oxford, UK). Datasets were indexed using either MOSFLM (Battye et al., 2011) or XDS (Kabsch, 2010), and scaled using the program SCALA (Evans, 2006) within the CCP4 software suite (Winn et al., 2011). The structures of the Y220C-ligand complexes were then solved using difference Fourier methods (rigid body refinement using PDB: 2J1X as a starting model), and refined using iterative cycles of manual model building in Coot (Emsley et al., 2010) and refinement with PHENIX (Adams et al., 2010). In the complex with compound **6**, the small molecule could only be modeled for one of the two chains in the asymmetric unit (chain B). Compound **11** was observed only in chain A and refined with partial occupancy. In this case, loop residues 219–224 in chain A were refined in two alternative conformations corresponding to the bound and unbound states. In the structure with **9**, there was significant negative difference density at the center of the iodine atom that is not involved in halogen bonding and significant positive difference density extending from the iodine into subsite 2, suggesting radiation damage and breakage of the C-I bond during data collection. X-Ray data collection and refinement statistics are given in Table 1. Structural figures were prepared using PyMOL (www.pymol.org).

### Molecular Dynamics Simulations

MD calculations were performed with the AMBER 12 suite of programs using three different starting structures of the Y220C mutant (PDB: 2J1X, 3ZME, and 4AGL). Protein parameters were taken from parm12SB, a modified version of the Cornell force field (Cornell et al., 1995, Hornak et al., 2006), and zinc parameters were taken from Bradbrook et al. (1998). Long-range electrostatic interactions were computed using the particle mesh Ewald method (Darden et al., 1993), and covalent bonds to hydrogen atoms were constrained by the SHAKE algorithm (Ryckaert et al., 1977). The time step was set to 2 fs with a non-bonded cutoff of 9 Å. Structural waters and ligands were removed before the start of the simulations, and the protein was placed in a truncated octahedral TIP4Pew water box with periodic boundary conditions (Horn et al., 2004). The box boundaries were at least 12 Å away from protein atoms. Chlorine ions were added to maintain electroneutrality of the system. The system was then equilibrated with 1,000 steps of minimization, followed by heating to 300 K during 10 ps of canonical ensemble (NVT) MD and by finally relaxing the pressure to 1 bar during 5 ns of isothermal-isobaric ensemble (NPT) MD to ensure correct water distribution close to the protein surface and at the box boundaries. Harmonic restraints with force constants of 5 kcal mol^−1^ Å^−2^ were applied to all protein atoms. These restraints were then gradually reduced to zero during 500 ps of NVT MD, followed by 10 ns of equilibration. A production run of 100 ns followed. The same procedure was repeated four times for each starting structure with different starting velocities to improve sampling. Only 50-ns-long production runs were performed for these repeat runs. In total, 900 ns (300 ns for each starting structure) were analyzed.

## Author Contributions

A.C.J. and A.R.F. designed the project; A.C.J., M.R.B., R.W., M.G.J.B, H.H., F.M.B., and J.S. designed and/or screened compounds; M.G.J.B. and J.S. synthesized compounds; M.R.B., R.W., F.M.B., and H.H. performed binding studies and analyzed data; A.C.J. determined and analyzed crystal structures, T.E.E. performed MD simulations; A.C.J. wrote the paper, with input from all authors.

## Figures and Tables

**Table 1:** X-Ray Data Collection and Refinement Statistics of p53-Y220C Ligand Structures

Compound	2	5	6	9	10	11	12
**Data Collection**							

Space group	*P*2_1_2_1_2_1_	*P*2_1_2_1_2_1_	*P*2_1_2_1_2_1_	*P*2_1_2_1_2_1_	*P*2_1_2_1_2_1_	*P*2_1_2_1_2_1_	*P*2_1_2_1_2_1_
*a* (Å)	64.99	64.89	64.82	65.22	65.01	64.91	65.16
*b* (Å)	71.06	71.11	71.18	71.11	70.99	71.24	71.27
*c* (Å)	105.03	104.94	104.81	105.35	105.18	105.02	105.11
Molecules/AU	2	2	2	2	2	2	2
Resolution (Å)^a^	29.6–1.36 (1.43–1.36)	29.5–1.62 (1.71–1.62)	29.5–1.78 (1.88–1.78)	29.5–1.47 (1.55–1.47)	29.5–1.35 (1.42–1.35)	35.4–1.50 (1.58–1.50)	43.7–1.74 (1.83–1.74)
Unique reflections	103,976	61,994	46,975	83,461	103,354	78,962	49,879
Completeness (%)^a^	99.2 (98.6)	99.4 (98.8)	99.6 (99.4)	99.4 (99.4)	96.5 (94.2)	99.8 (99.6)	98.2 (96.7)
Multiplicity^a^	5.4 (5.4)	6.0 (5.9)	4.9 (4.9)	5.4 (5.4)	6.1 (5.9)	5.6 (5.3)	5.6 (5.4)
*R*_merge_ (%)^a^	5.4 (59.5)	8.6 (54.3)	5.1 (63.0)	6.0 (55.7)	6.3 (46.3)	7.3 (33.5)	7.0 (34.9)
Mean *I*/σ*I*^a^	15.2 (3.7)	14.5 (3.3)	16,9 (3.2)	13.4 (3.1)	15.5 (3.7)	14.0 (4.1)	14.9 (4.7)
Wilson *B* value (Å^2^)	13.0	14.3	22.8	14.0	11.2	12.7	16.3

**Refinement**							

*R*_work_ (%)^b^	14.3	17.8	17.8	14.9	14.5	13.9	16.9
*R*_free_ (%)^b^	16.6	20.3	20.6	17.8	16.9	16.9	19.1
No. of atoms							
Protein^c^	3187	3129	3113	3135	3180	3217	3104
Zinc	2	2	2	2	2	2	2
Water	425	480	334	406	566	554	440
Ligands	70	46	23	41	49	13	64
RMSD bonds (Å)	0.006	0.006	0.007	0.006	0.006	0.006	0.007
RMSD angles (°)	1.0	1.0	1.0	1.0	1.1	1.0	1.0
Mean *B* (Å^2^)	21.0	17.8	31.4	21.0	16.5	18.0	21.2
PDB entry	5AB9	5ABA	5AOI	5AOJ	5AOK	5AOL	5AOM

RMSD, root-mean-square deviation.

a Values in parentheses are for the highest-resolution shell.

b *R*_work_ and *R*_free_ = ∑||*F*_obs_| − |*F*_calc_||/∑|*F*_obs_|, where *R*_free_ was calculated with 5% of the reflections chosen at random and not used in the refinement.

c Number includes alternative conformations.

**Table 2:** Dissociation Constants of p53-Y220C Ligand Complexes

Compound	*K*_D_ (μM)^a^
**1**	125 ± 10 (ITC)^b^
**2**	470 ± 90 (NMR)
**3**	9.7 (ITC)^c^
**4**	820 ± 70 (NMR)^c^
**5**	1,040 ± 110 (NMR)^c^
**6**	940 ± 110 (NMR)
**7**	2,400 ± 300 (NMR)
**8**	>10,000 (NMR)
**9**	21 (ITC)
**10**	>2,000 (NMR)
**11**	4,800 ± 900 (NMR)
**12**	1,270 ± 570 (NMR)
**13**	ND

a The method used for determination of the dissociation constant is given in parentheses: nuclear magnetic resonance spectroscopy (NMR) or isothermal calorimetry (ITC). ND, not determined.

b Data taken from Boeckler et al. (2008).

c Data taken from Wilcken et al. (2012a).

## Accession Numbers

The atomic coordinates of the p53 Y220C mutant crystal structures have been deposited in the PDB, www.pdb.org (PDB: 5AB9, 5ABA, 5AOI, 5AOJ, 5AOK, 5AOL, and 5AOM).
